# Scientific Advancements in Drug Development and Trials for Urothelial Carcinoma: Insights From the 2023 ASCO-GU Cancers Symposium

**DOI:** 10.14336/AD.2023.0502

**Published:** 2023-12-01

**Authors:** Dechao Feng, Dengxiong Li, Ruicheng Wu, Ping Han

**Affiliations:** Department of Urology, Institute of Urology, West China Hospital, Sichuan University, Chengdu 610041, China

**To the editor**,

Aging is related to cancer, and an increasing incidence of malignant tumors among the older population is observed [1]. Urinary tumors, such as prostate cancer, bladder cancer (BC), and renal cancer, frequently occur in the aging population [2-10]. Urothelial carcinoma (UC) ranks as the fourth most common tumor, with an estimated 160,000 new diagnoses and 31,000 deaths each year [11]. Due to the rarity of upper tract UC (5%-10%), high-level evidence is often scarce to provide strong recommendations, and in many cases, guidelines are extrapolated from existing evidence on bladder UC (90%-95%) [12, 13]. Muscle-invasive BC (MIBC) accounts for about 25% of newly diagnosed BC patients [14]. Non-MIBC (NMIBC) patients usually undergo transurethral resection followed by intravesical chemotherapy or Bacillus Calmette-Guérin (BCG) therapy, while MIBC cases are usually treated with radical cystectomy (RC) plus pelvic node dissection [6, 14-16]. However, MIBC is prone to relapse after RC, and only about 5% of metastatic cancer patients survive for at least 5 years post-diagnosis [17]. Thus, metastasis is one of the most important causes of death for such patients.

The standard of care for the treatment of metastatic UC for more than 20 years has been cisplatin-based combinations [18]. However, a number of sizable studies that looked at the effectiveness of immunotherapy employing immune checkpoint inhibitors (ICIs) have cast doubt on this time-honored strategy. Moreover, immunotherapy has shown significant progress in other solid tumors, such as lung cancer [19]. We aimed to summarize the many novel agents and trials for UC presented at the 2023 ASCO-GU Cancers Symposium.

In terms of presented novel agents and trials for UC in this congress, PD-1/PD-L1-based ICIs (Pem-brolizumab, Atezolizumab, Nivolumab, and Avelumab), platinum-based chemotherapy (PCT), antibody-drug conjugates (ADCs), (Enfortumab vedotin (EV) and Sacituzumab govitecan (SG)), and fibroblast growth factor receptor kinase inhibitors (Erdafitinib and Derazantinib) were most presented (Fig. 1A-B). UC patients mainly focused on locally advanced or metastatic UC (la/mUC), NMIBC, and MIBC (Fig. 1C). We summarized these trials in Supplementary Table 1 and described several interesting trials here.

EV-103 Cohort K (NCT03288545) evaluated first-line EV and Pembrolizumab or EV alone in patients with la/mUC who were cisplatin-ineligible. EV and Pembrolizumab led to EV dose reduction and showed a clinically meaningful objective response rate (ORR: 64.5%; 95% CI, 52.7-75.1) with a manageable safety profile compared to EV alone (45.2%; 95% CI: 33.5-57.3). Moreover, EV and Pembrolizumab contributed to clinically meaningful reductions in quality of life (QoL). Based on these results, three Phase 3 trials (NCT04223856, NCT04700124, NCT03924895) are being conducted for EV and Pembrolizumab in the first-line la/mUC and MIBC patients.

IMVIGOR 130 phase III trial (NCT02807636) reported final overall survival (OS) data, showing that improved OS with Atezolizumab plus platinum/ gemcitabine vs placebo plus platinum/gemcitabine did not reach statistical significance in patients with la-/mUC. Two large Cohort trials (Javelin Bladder100 (NCT02603432) and NCT04822350) supported the usage of Avelumab as maintenance therapy in la/mUC patients without progression after PCT due to benefits of OS. In this meeting, CheckMate 274 trial (NCT02632409) reported extended follow-up data, indicating that Nivolumab continued to show disease-free survival (DFS), non-urothelial tract recurrence-free survival, and distant metastasis-free survival benefits versus placebo.

**Figure 1.**
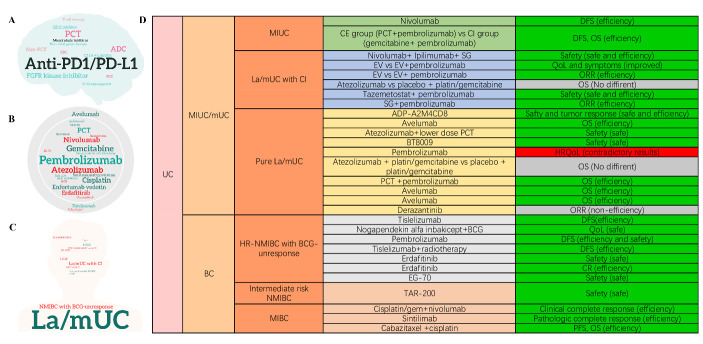
**Novel agents and trials for UC**. (**A**) word cloud showing medication clusters; (**B**) word cloud showing specific treatments; (**C**) word cloud showing patient types; (**D**) summary of novel agents and trials for UC in this meeting. UC: urothelial carcinoma; MIBC: muscle-invasive bladder cancer; BCG: Bacillus Calmette Guérin; EV: enfortumab vedotin; la/mUC: locally advanced or metastatic UC; SG: Sacituzumab govitecan PCT: platinum-based chemotherapy; ORR: objective response rate; MIUC: muscle-invasive urothelial carcinoma; PFS: progression-free survival; OS: overall survival; HR: high-risk. QoL: quality of life; HRQoL: health-related QoL.

Cohort B of KEYNOTE-057 (NCT02625961) reported that Pembrolizumab monotherapy showed notable antitumor activity in patients with BCG-unresponsive non-CIS papillary high-risk (HR) NMIBC after ~45 months of follow-up (12-month DFS rate: 43.5 (34.9-51.9); 12-month OS rate: 96.2 (91.1-98.4)). FIDES-02 study (NCT04045613) explored the activity of Derazantinib in patients with mUC and FGFR1-3 genetic aberrations. Confirmed ORR was 8.2% (95% CI 2.2, 19.6) and the disease control rate was 28.6% (95% CI: 16.6, 43.3). Other emerging agents showed various promising results and deserve expectation in the future. The drugs and trials were presented in Figure 1D.

For trials in progress, researchers still mainly concentrate on patients with mUC, HR BCG-unresponsive NMIBC or MIBC. What's different from the published trials is that researchers are commencing on investigating the effect of ADCs with or without immunotherapy on such patients, suggesting that ADCs are receiving more attention. The ongoing trials are summarized in Table 1.

Median OS for advanced UC patients undergoing PCT followed by immunotherapy is still smaller than 1 year and there is an urgent need for alternative therapies[20]. With the advent of next-generation sequencing, The Cancer Genome Atlas project reported comprehensive molecular alterations of high-grade MIUC and found that numerous genomic aberrations, such as FGFR3 (15%), HER2 (8%), and EGFR (6%)[20-22]. The presence of multiple oncogenic alterations in UC, together with the low efficacy of first-line immunotherapy in this scenario, suggests that oncogenic alterations may have potential as a predictive biomarker for therapeutic decision-making. In this case, ADCs may offer new avenues for biomarker-driven treatment in advanced UC, especially for patients with oncogenic alterations [22]. In addition, rapid advancements have taken place in gene therapy technology and gene therapy that targets genes related to aging represents an exciting research direction with tremendous potential[23]. Nanomedicines have been widely studied in cancer therapy in recent years [24].

Based on the current evidence and the advancements in this meeting, we proposed the following management strategy of UC. PCT is the first-line standard therapy for all patients who are candidates for either cisplatin or carboplatin. Patients positive for PD-L1 and ineligible for cisplatin may receive immunotherapy (Atezolizumab or Pembrolizumab) or EV and Pembrolizumab, among which Erdafitinib could be considered in la/mUC patients with FGFR alterations. If the disease does not progress after PCT, maintenance immunotherapy (Avelumab) is suggested. Immuno-therapy (Pembrolizumab) is the recommended second-line therapy for patients who do not have maintenance therapy. Later-line treatments like Derazantinib, EZH2 inhibitor (Tazemetostat), T-cell therapy (ADP-A2M 4CD8) and non-viral gene therapy (EG-70) could be tried in specific cases with the patient’s consent. Pembrolizumab monotherapy might be recommended in patients with HR NMIBC unresponsive to BCG who declined or were ineligible to undergo radical resection. In future trials, oncogenic alterations can be considered in trial designs and PCT-based target therapy or ADCs with immunotherapy should be conducted in more mUC patients or MIBC candidates for RC.

**Table 1:** Trials in progress for UC.

Author	Country	Clinical trials	Patients	Therapeutic regimen	Patient feature
**Po-Jung Su et al.**	Asia-Pacific (APAC) region	NA	286	Avelumab first-line (1L) maintenance after 1L platinum-based chemotherapy	Unresectable locally advanced or metastatic (stage IV) measurable UC
**Massimo Lazzeri et al.**	Italy	(EudraCT 2021-003751-42_studio ICH-013 (MMC))	160	Neoadjuvant MMC vs standard of care (RCT)	Primary treatment-naive NMIBC
**Christopher Gaffney et al.**	USA	(NCT04179162)	25	Gemcitabine and BCG	BCG-relapsed/BCG-exposed NMIBC
**Sarmad Sadeghi et al.**	USA	(NCT04579224)	465	Randomized 3 arm study comparing eribulin vs. gemcitabine plus eribulin vs. SOC (docetaxel, paclitaxel, or gemcitabine)	Metastatic UC refractory to or ineligible for PD/PDL1 antibody (Ab)
**Ashish M. Kamat et al.**	International	(NCT05014139)	NA	Intravesical enfortumab vedotin	High-risk BCG-unresponsive NMIBC
**Neal D. Shore et al.**	International	(NCT02625961)	60	Pembrolizumab and vibostolimab vs pembrolizumab and favezelimab (RCT)	High-risk BCG-unresponsive NMIBC
**Guru P. Sonpavde et al.**	USA	(NCT05574504)	36	Lurbinectedin plus avelumab as maintenance therapy	Metastatic UC with stable or responding disease following platinum-based chemotherapy
**Himanshu Nagar et al.**	USA	(NCT04936230)	144	Atezolizumab versus atezolizumab and radiation therapy (RCT)	Platinum-ineligible/refractory metastatic UC
**Andrea Necchi et al.**	International	(NCT03924895)	857	Neoadjuvant pembrolizumab plus RC+PLND plus adjuvant pembrolizumab vs RC+PLND vs Neoadjuvant pembrolizumab+enfortumab vedotin plus RC+PLND plus adjuvant pembrolizumab+enfortumab vedotin (RCT)	Cisplatin ineligible or decline cisplatin-based treatment with treatment-naive MIBC
**Evan Y. Yu et al.**	International	(NCT04579380)	30	Tucatinib and trastuzumab	HER2+ locally advanced or metastatic disease, with progression during or after, or intolerance of, the most recent line of systemic therapy.
**Christopher J et al.**	International	(NCT04700124)	784	Neoadjuvant enfortumab vedotin + pembrolizumabo plus RC+PLND plus adjuvant enfortumab vedotin+ pembrolizumab vs neoadjuvant chemotherapy [gemcitabine + cisplatin] plus RC+PLND) (RCT)	Cisplatin-eligible patients with MIBC
**Jean H. Hoffman-Censits et al.**		(NCT05312671)	17-63	Neoadjuvant atezolizumab+etoposide+investigator choice cisplatin or carboplatin chemotherapy+ cystectomy+ adjuvant atezolizumab	Localized small cell neuroendocrine bladder cancer
**Thomas Powles et al.**	International	(NCT03547973)	158	Sacituzumab govitecan plus zimberelimab vs avelumab vs zimberelimab (RCT)	Cisplatin-eligible patients with unresectable or metastatic UC
**Ignacio Duran et al.**	International	(NCT03547973)	226	Sacituzumab govitecan (SG), SG plus zimberelimab (ZIM), SG plus ZIM plus domvanalimab, or carboplatin + gemcitabine (RCT)	Cisplatin ineligible patients with treatment-naive metastatic UC
**Benjamin Garmezy et al.**	USA	(NCT05242822)	120	KIN-3248, a next-generation, irreversible pan-FGFR inhibitor	Advanced UC harboring FGFR2 and/or FGFR3 gene alterations.
**Thomas Powles et al.**	International	(NCT04879329)	NA	Disitamab vedotin with or without pembrolizumab	HER2-expressing UC
**Andrea Necchi et al.**	International	NA	30	Neoadjuvant nivolumab + visugromab vs nivolumab + Placebo	MIBC
**Seth P. Lerner et al.**	USA	(NCT05483868)	23	Intramural injection - belzupacap sarotalocan vs intratumoral injection - belzupacap sarotalocan+Laser vs post-injection cystectomy vs post-Injection TURBT	NMIBC
**Gopa Iyer et al.**	USA	(NCT05103358)	NA	Albumin-bound (nab)-sirolimus, a novel mTOR inhibitor (mTORi)	Patients with alterations in TSC1 (Arm A) and TSC2 (Arm B).

IL: first line; RCT: randomized controlled trials; NMIBC: non-muscle-invasive bladder cancer; MIBC: muscle-invasive bladder cancer; TURBT: transurethral resection of bladder tumor; UC: urothelial carcinoma; RC: radical cystectomy; PLND: pelvic lymph node dissection; MMC: mitomycin C. BCG: Bacillus Calmette Guérin.

## Supplementary Materials

The Supplementary data can be found online at: www.aginganddisease.org/EN/10.14336/AD.2023.0502.


