# Supplementary material for: Scientific Advancements in Drug Development and Trials for Urothelial Carcinoma: Insights From the 2023 ASCO-GU Cancers Symposium
Source: Aging Dis. 2023 Dec 1;14(6):1953–7. doi: 10.14336/AD.2023.0502 (PMC10676780; doi:10.14336/AD.2023.0502)
Supplement: Supplementary file 1 [file AD-14-6-1953-s.pdf]

SUPPLEMENTARY DATA

# Scientific Advancements in Drug Development and Trials for Urothelial Carcinoma: Insights From the 2023 ASCO-GU Cancers Symposium

**Dechao Feng, Dengxiong Li, Ruicheng Wu, Ping Han**

© 2023. Feng DC et al. Published online at http://www.aginganddisease.org/EN/10.14336/AD.2023.0502

# SUPPLEMENTARY DATA

**Supplementary Table 1.** Specifying novel agents and trials for UC.

| Author | Country | Registration number | Patients number | Medicine | Patient feature | Main end-points | Main results |
|---|---|---|---|---|---|---|---|
| Matthew I. Milowsky et al. | International | LBA439 (NCT03288545) | 152 | EV vs EV+ pembrolizumab | CI patients with la/mUC. | QoL and symptoms | This study showed that EV+ pembrolizumab in cisplatin-ineligible patients with la/mUC was associated with preservation or improvement of QoL, functioning, and symptoms. |
| Matt D. Galsky et al. | International | LBA440 (NCT02807636) | 851 | Atezolizumab + plt/gem vs placebo + plt/gem | Patients with la/mUC | OS | There was no difference in OS between the two groups. |
| Aristotelis Bamias et al. | International | LBA441 (NCT02807636) | 719 | Atezolizumab monotherapy vs placebo + plt/gem | Patients with la/mUC | OS | Only in the PD-L1–high mUC subgroup, patients accepted Atezolizumab monotherapy had better OS than these received placebo + plt/gem. |
| Andrea Necchi et al. | International | LBA442 (NCT02625961) | 132 | Pembrolizumab (q3W) for <=35 cycles | Patients with BCG-unresponsive HR non-muscle-invasive bladder cancer (NMIBC) with papillary tumors only (high grade Ta or any-grade T1) at baseline and ECOG PS 0-2. | DFS in 12 months | Results suggest patients with non-CIS papillary HR NMIBC unresponsive to BCG who declined or were ineligible to undergo RC may also benefit from pembrolizumab monotherapy. |
| Matt D. Galsky et al. | International | LBA443 (NCT02632409) | 353 | Nivolumab (240 mg q2W) versus placebo | Patients with HR MIUC after radical resection. | DFS | Nivolumab continued to show DFS benefits versus placebo. |
| Matt D. Galsky et al. | International | LBA447 (NCT03558087) | 76 | Four cycles of gemcitabine, cisplatin, plus nivolumab | Patients with cisplatin-eligible with cT2-T4aN0M0 urothelial bladder cancer. | cCR | Transurethral resection of bladder tumor followed by gemcitabine, cisplatin, plus nivolumab achieves stringently defined cCR in a substantial subset of patients with MIBC. >=2-year bladder-intact survival is achieved in the majority of patients with a cCR |
| Jason Brown et al. | International | LBA448 (NCT02365766) | 82 | CE group (gemcitabine +cisplatin +pembrolizumab) vs CI group (gemcitabine+ pembrolizumab) | Patients were surgical candidates with clinical stage T2-4aN0M0 MIUC. | DFS, OS | Neoadjuvant chemoimmunotherapy demonstrated significant down staging in CE and CI MIUC pts prior to definitive surgery, meeting the primary endpoint. Survival correlated with pathologic response. |
| Yuki Endo et al. | Japan | Post session 452 | 70 | PCT +pembrolizumab | Patients with mUC received PCT followed by pembrolizumab. | OS | This study reported that early introduction of pembrolizumab may be associated with longer OS in the patients with mUC who are resistant to chemotherapy. |
| Lorenzo Antonuzzo et al. | Italian | LBA469 (NCT02603432) | 464 | Avelumab 800 mg every two weeks | Patients with locally advanced or metastatic urothelial carcinoma | OS | This study supports for avelumab as a standard of care in eligible pts |

© 2023. Feng DC et al. Published online at http://www.aginganddisease.org/EN/10.14336/AD.2023.0502

| | | | | | | | |
|---|---|---|---|---|---|---|---|
| | | | | | without progression after PCT. | | with locally advanced or metastatic urothelial carcinoma. |
| Philippe Barthelemy et al. | France | LBA471 (NCT04822350) | 591 | Avelumab | Patients with locally advanced urothelial carcinoma without progression after PCT. | OS | The study confirms the clinical activity and acceptable safety profile of avelumab in a heterogeneous population |
| Soufyan Annakib et al. | France | LBA490 (NCT03584659) | 39 | Pembrolizumab | Patients with la/mUC | HRQoL | HRQoL for la/mUC patients after first pembrolizumab infusion showed contradictory results with previous reports with a trend for HRQoL degradation after one treatment cycle. |
| AmarnathChallapalli et al. | United Kingdom | LBA493 (NCT01616875) | 26 | Cabazitaxel +cisplatin | Patients with MIBC were included if fit to receive NAC and to undergo RC. | PFS, OS | Neoadjuvant cabazitaxel with cisplatin chemotherapy is an effective regimen with 65.4% patients alive and progression-free at 5 years. |
| Karim Chamie et al. | USA | LBA495 (NCT03022825) | 86 | Nogapendekin alfa inbakicept+BCG | Patients with BCG-unresponsive CIS with or without Ta/T1 disease. | QoL | QOL measurement supports good tolerability of the intravesical Nogapendekin alfa inbakicept plus BCG in BCG-unresponsive, high-grade NMIBC patients with CIS. |
| Capucine Baldini et al. | International | LBA498 (NCT045613622) | 49 | BT8009 | Patients with advanced malignancies. | Safety | BT8009 was well tolerated and demonstrated promising preliminary antitumor activity |
| Peter H. O'Donnell et al. | International | LBA499 (NCT03288545) | 149 | EV vs EV+ pembrolizumab | CI patients with la/mUC. | ORR | EV+ pembrolizumab showed promising ORR in CI patients with la/mUC. |
| Andrea Necchi et al. | International | LBA501 (NCT04045613) | 282 | Derazantinib 300 mg qd | Patients with advanced mUC. | ORR | Derazantinib showed signals of clinical activity in some patients with mUC and FGFR1-3 genetic aberrations but the observed ORR and PFS. |
| Guillermo de Velasco et al. | Spain | LBA502 (NCT04602078) | 82 | Atezolizumab | Patients with la/mUC considered unfit for full dose of PCT. | ORR, PFS, OS | Atezolizumab with split doses of PCT was safely administered in a population of frail patients with mUC who were unfit for PCT showing promising preliminary survival outcomes in terms of response. |
| James W.F.Catto et al. | International | LBA503 (NCT04172675) | 10 | Erdafitinib 6 mg once daily | Patients with BCG unresponsive HR NMIBC with FGFR3/2alt and CIS. | Safety | Erdafitinib is efficacy in patients with HR-NMIBC with FGFRalt. |
| Siamak Daneshmand et al. | International | LBA504 (NCT04172675) | 10 | Erdafitinib 6 mg once daily | Patients with BCG unresponsive HR NMIBC with intermediate-risk and FGFRalt, no CIS. | CR | This study demonstrates efficacy of erdafitinib in adult patients with intermediate-risk NMIBC with FGFRalt. |

© 2023. Feng DC et al. Published online at http://www.aginganddisease.org/EN/10.14336/AD.2023.0502

| | | | | | | | |
|---|---|---|---|---|---|---|---|
| F.Johannes P. van Valenberg et al. | Netherlands | LBA505 (NCT02720367) | 12 | TAR-200 | Patients with a papillary recurrence after prior histologically proven, intermediate risk NMIBC. | Safety | TAR-200 appears to be safe and well tolerated in patients with intermediate-risk NMIBC. |
| Maha H. A. Hussain et al. | International | LBA506 (NCT03854474) | 24 | Tazemetostat 800 mg bid+ pembrolizumab 200 mg every 3 weeks | Patients with cisplatin-refractory or cisplatin/chemo-ineligible advanced urothelial cancer. | Making sure the recommended phase two dose | The recommended phase two dose for tazemetostat is 800 mg + 200 mg Pembrolizumab q 3 weeks. |
| Srikala S. Sridhar et al. | International | LBA508 (NCT02603432) | 700 | Avelumab maintenance + best supportive care vs best supportive care alone | Patients with unresectable la/mUC that did not progress after accepting PCT. | OS | Long-term follow-up confirms that avelumab maintenance provides similar OS and PFS benefits in patients with advanced UC who are progression free following standard-of-care cisplatin- or carboplatin-based chemotherapy, with an acceptable safety profile. |
| Fangning Wan et al. | China | LBA509 (ChiCTR2000032730) | 15 | Sintilimab 200 mg | Patients were cT2-T4 N0 MIBC and were considered eligible for RC. | Pathologic complete response | Neoadjuvant sintilimab was feasible and provided meaningful pathologic responses in patients with MIBC. |
| Zhiyong Li et al. | China | LBA510 (ChiCTR2000035275) | 14 | Tislelizumab 200 mg | Patients with HR NMIBC BCG-unresponsive papillary tumors. | DFS | Our preliminary results supported the use of tislelizumab combined with radiotherapy as a promising bladder-preserving therapy for patients with BCG-unresponsive HR NMIBC who were ineligible for or refused RC. |
| Gary D. Steinberg et al. | Canada | LBA512 (NCT04752722) | 19 | EG-70 | Patients with BCG-unresponsive CIS NMIBC. | SD or CR | These results demonstrate the safety, tolerability and durable therapeutic potential of intravesical EG-70 in patients with BCG-unresponsive CIS. |
| David H Aggen et al. | International | LBA517 (NCT04044859) | 43 | ADP-A2M4CD8 | Patients with advanced UC. | Safety | ADP-A2M4CD8 continues to show an acceptable benefit to UC. |
| Rohit K. Jain et al. | Orlando | LBA521 (NCT04863885) | 9 | Nivolumab+ Ipilimumab+ SG | Patients with CI mUC | Safety | The RP2D of SG was identified as 8mg/kg in combination with ipilimumab 3 mg/kg+ Nivolumab 1 mg/kg as first-line therapy for CI mUC. |
| Petros Grivas et al. | International | LBA518 (NCT03547973) | / | SG+pembrolizumab | Patients with mUC that progressed after PCT | ORR | SG plus pembrolizumab demonstrated a high ORR with a manageable safety profile in mUC in patients who progressed after PCT. |

la/mUC: locally advanced or metastatic urothelial cancer; EV: Enfortumab vedotin; QoL: quality of life; OS: Overall survival; plt/gem: platinum/gemcitabine; DFS: Disease-free survival; RC: radical cystectomy; cCR: clinical complete response; CE: Cisplatin eligible; CI: cisplatin ineligible; MIUC: muscle-invasive urothelial carcinoma; PCT: platinum-based chemotherapy; HRQoL: Health-related quality of life; PFS: Progression-free survival; MIBC: muscle-invasive bladder cancer; NAC: neoadjuvant chemotherapy; ORR: objective response rate.

© 2023. Feng DC et al. Published online at http://www.aginganddisease.org/EN/10.14336/AD.2023.0502
